# Injectable Contraceptives Differentially Affect Select CD4+ HIV‐1 Target Cells in the Genital Tract but Not Systemically: Implications for HIV‐1 Acquisition

**DOI:** 10.1111/aji.70093

**Published:** 2025-05-11

**Authors:** Chanel Avenant, Alexis J. Bick, Johnson M. Moliki, Sigcinile Dlamini, Michele Tomasicchio, G. Justus Hofmeyr, Charles Morrison, Pai‐Lien Chen, Janet P. Hapgood

**Affiliations:** ^1^ Department of Molecular and Cell Biology University of Cape Town Cape Town South Africa; ^2^ Centre for Lung Infection and Immunity Division of Pulmonology Department of Medicine University of Cape Town and UCT Lung Institute Cape Town South Africa; ^3^ South African MRC Centre for the Study of Antimicrobial Resistance University of Cape Town Cape Town South Africa; ^4^ Effective Care Research Unit Eastern Cape Department of Health Universities of the Witwatersrand and Fort Hare East London South Africa; ^5^ Walter Sisulu University East London South Africa; ^6^ Department of Obstetrics and Gynecology University of Botswana Gaborone Botswana; ^7^ Independent Consultant Chapel Hill North Carolina USA; ^8^ Family Health International (FHI) 360 Durham North Carolina USA; ^9^ Institute of Infectious Disease and Molecular Medicine University of Cape Town Cape Town South Africa

**Keywords:** CD4, DMPA, female genital tract, HIV‐1 acquisition, injectable contraceptives, NET‐EN, PBMCs

## Abstract

**Problem:**

Observational data suggest lower HIV susceptibility in women using the injectable contraceptive norethisterone enanthate (NET‐EN) versus intramuscular depo medroxyprogesterone acetate (DMPA‐IM). Clinical data investigating the effects of injectables on HIV target cells are inconsistent or limited. No data on HIV target cells are available from head‐to‐head randomized trials comparing DMPA‐IM and NET‐EN, nor at peak progestin concentrations.

**Method of Study:**

The women's health, injectable contraception, and HIV (WHICH) trial randomized women to DMPA‐IM or NET‐EN at two South African sites (2018–2019). Cells from blood and cytobrushes from women at one site, taken at baseline and 1 week post the 24‐week injection (at peak progestin levels), were analyzed by flow cytometry for select HIV‐1 target cells (CD4^+^ cells expressing HIV‐1 co‐receptors, an integrin and/or activation markers).

**Results:**

Systemically, DMPA‐IM and NET‐EN similarly reduced the frequency and number of some CD4^+^ cells and expression of some CD4^+^ cell surface markers. In contrast, female genital tract (FGT) results showed significantly different cell numbers between contraceptives for most cell populations; DMPA‐IM tended to increase, but NET‐EN tended to decrease cell numbers. Excluding for non‐study progestin use revealed significant increases in frequency and/or number of several FGT cell populations from baseline to 25 weeks, within the DMPA‐IM arm.

**Conclusions:**

Both contraceptives exert minimal effects on systemic CD4^+^ cells but have differential effects in the FGT. The changes in frequency and numbers of HIV‐1 target cells investigated, particularly after exclusion for non‐study progestin use, suggest that DMPA‐IM use may increase HIV‐1 acquisition in the FGT compared to NET‐EN use.

## 1 Introduction

Young women in sub‐Saharan Africa accounted for 77% of new HIV‐1 infections worldwide in 2022 [1], where injectable progestin‐only contraceptives are the most widely used birth control methods [2]. The three‐monthly intramuscular injectable depo medroxyprogesterone acetate (DMPA‐IM) is the most commonly used hormonal contraceptive in sub‐Saharan Africa, while the two‐monthly intramuscular injectable norethisterone (NET) enanthate (NET‐EN) is widely used in South Africa [3]. In some areas of South Africa, injectables are used interchangeably, assuming that they both have no risks and are classified by the World Health Organization as having no restrictions for use. However, limited observational data suggest that DMPA‐IM, compared to NET‐EN, is associated with a 32%–40% increased susceptibility to HIV‐1 acquisition [4, 5]. Compared to women not using any hormonal contraceptive or limited use of condoms‐only, DMPA‐IM users may have a 40%–50% increased risk of HIV‐1 acquisition, while limited data do not suggest increased vulnerability to HIV‐1 acquisition for NET‐EN [4, 6–9]. The evidence for contraceptive options and HIV outcomes (ECHO) trial, which assessed HIV‐1 susceptibility in women randomized to DMPA‐IM, a copper intrauterine device or levonorgestrel subdermal implant [10] did not detect differences in HIV susceptibility amongst the methods evaluated. However, the study was powered to detect only a ≥50% increase in HIV incidence across study arms and did not include a NET‐EN comparator or a control group. Thus, the question of relative risks of DMPA‐IM versus NET‐EN use on HIV‐1 acquisition and the underlying biological mechanisms remains unanswered.

Several studies have proposed that cell numbers, frequencies and expression levels of markers on CD4^+^ cell populations in the female genital tract (FGT) are likely an important factor driving HIV‐1 acquisition and are used as a proxy for HIV‐1 infectivity [11, 12, 13, 14, 15]. CD4^+^ cells are the main target of HIV‐1 infection, with the CD4 receptor along with chemokine co‐receptors CCR5 and CXCR4 serving as HIV‐1 entry receptors. CD25, CD38, CD69, and HLA‐DR are cellular markers of T‐cell activation, and their expression on CD4^+^ cells renders them highly susceptible to HIV‐1 infection both in vivo and in vitro *[* 12, 16–18]. The integrin α4β7 is a T‐cell mucosal homing receptor that also facilitates cell‐to‐cell spread of HIV‐1 infection in tissues [19, 20, 21].

Several in vitro and clinical studies have investigated the individual and comparative effects of medroxyprogesterone (MPA) or DMPA‐IM and NET or NET‐EN, respectively, on systemic and FGT immune cell markers. In vitro studies suggest differential effects between MPA and NET, both in the FGT and blood [22, 23]. Whether these occur in vivo remains unclear. Although there are no flow cytometry data from randomized clinical trials comparing DMPA‐IM and NET‐EN, there are many non‐randomized studies, particularly for DMPA‐IM, and only one randomized study reporting on DMPA‐IM effects on CD4^+^ HIV‐1 target cells expressing the co‐receptor CCR5 or activation markers or α4β7, or combinations thereof [24]. Immune cell data from many clinical trials are inconsistent. Previous studies report some increases [24, 25, 26, 27, 28, 29, 30, 31, 32, 33] and/or some decreases [24, 25, 27, 29, 31, 34, 35], while others do not detect any changes [24, 28, 35, 36] in CD4^+^ cell populations in the FGT. More limited systemic studies report some increases [14, 29, 30] and/or some decreases [14, 29, 30, 37] or no detected changes [13, 26, 34, 37] in cell populations in the blood. Only a few non‐randomized studies concurrently investigated the effect of injectables on immunological changes in both the FGT and blood, with varied results [26, 29, 30, 34, 38]. A prospective cohort study without disaggregating DMPA‐IM and NET‐EN use showed that injectable contraceptives increased CCR5 expression and CD4^+^ cell activation in the cervix but not the blood [26]. A parallel longitudinal cohort trial reported significantly decreased CD4^+^ cell numbers but no effect on CD4^+^CD69^+^ nor CD4^+^CCR5^+^ cells in the FGT 180 days after initiation of DMPA‐IM or NET‐EN, with no significant changes detected for limited systemic cell populations for either contraceptive [34]. A substudy from the ECHO trial reported increased frequency of FGT Th17‐like CD4^+^ T cells, including a highly susceptible activated population co‐expressing CD38, CCR5, and α4β7 in DMPA‐IM users, 1 month after a single injection compared to initiation [24].

Multiple confounding factors could contribute to variability in the clinical data, in particular the widespread misreporting of use of contraceptives not assigned or disclosed in trials [39, 40, 41, 42, 43, 44]. We previously reported on non‐study progestin use from the WHICH (women's health, injectable contraception, and HIV) trial which randomized women to DMPA‐IM or NET‐EN at study sites where DMPA‐IM is the most commonly used contraceptive method [40, 45]. We estimated that substantial misreporting of contraceptive use before and during the trial occurred, as well as some “tail” effects of injectable use before trial enrolment [40]. This was despite self‐reported absence of use of these injectables before (6 months for DMPA‐IM and 4 months for NET‐EN) or during the trial [40].

Here we sought to determine the effects of DMPA‐IM and NET‐EN after contraceptive initiation as well as their relative effects on CD4^+^ cells expressing HIV‐1 co‐receptors, select activation markers and an integrin, systemically and in the FGT, in samples from the WHICH trial. We chose specific markers based on their reported association with HIV‐1 acquisition and/or modulation by DMPA‐IM or NET‐EN use [14, 24, 26, 29–34]. We compared changes in the expression (median fluorescent intensity, MFI) of these cell surface markers, as well as the frequency and number of CD4^+^ cells expressing these markers using flow cytometry in both peripheral blood mononuclear cells (PBMCs) and cytobrush cells from women randomized to DMPA‐IM or NET‐EN, between samples collected at baseline (Day 0, D0) and at 25 weeks (25 W), a time corresponding to peak serum progestin levels [40]. We further investigated the effects of non‐study progestin use on the select HIV‐1 target cells.

## 2 Materials and Methods

### 2.1 Ethics and Biosafety

This is a secondary study of the open‐label randomized WHICH clinical trial [45] (PACTR 202009758229976 https://pactr.samrc.ac.za/Search.aspx). Ethics and biosafety approval for this secondary study was obtained from the University of Cape Town Human Research Ethics Committee (HREC 664/2018) and the University of Cape Town (IBC047‐2020), respectively.

### 2.2 Study Participants

Trial participants were randomized to intramuscular injectable contraceptive, 150 mg DMPA‐IM every 12 weeks (*n* = 262) or 200 mg NET‐EN every 8 weeks (*n* = 259), at two South African research sites in Durban and East London: MatCH Research Unit (MRU) and East London and Mdantsane public health clinics and hospitals [45]. Women were aged between 18 and 40 and seeking injectable contraception. Participants were excluded if they reported use of DMPA‐IM or NET‐EN in the previous 6 or 4 months, respectively, were pregnant, or were positive for HIV‐1 or sexually transmitted infections. Women were counselled on reducing HIV‐1 susceptibility and condom use. All participants provided written informed consent.

### 2.3 Sample Collection and Study Groups

**FIGURE 1:**
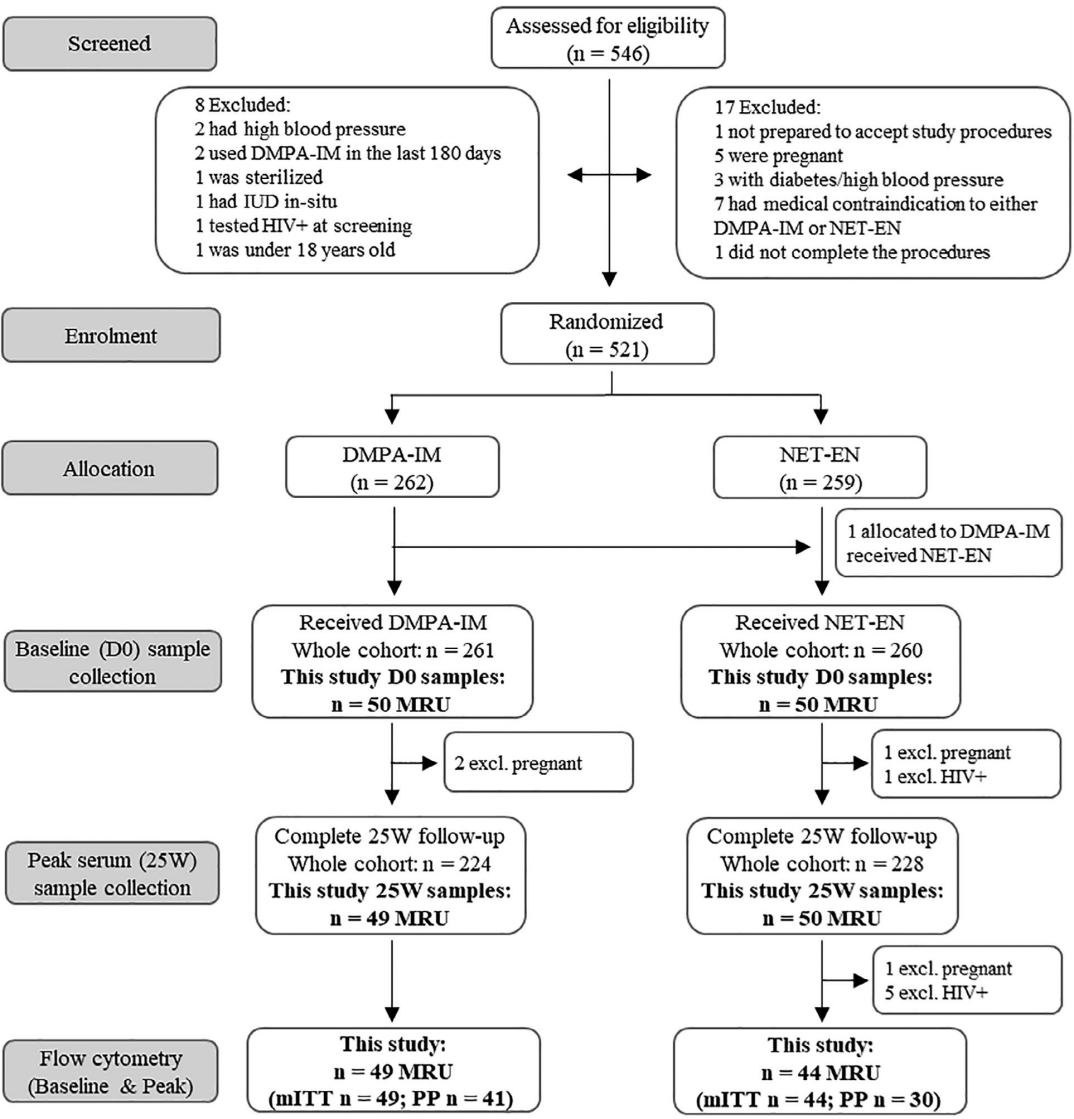
Consort flow diagram.

For a randomly selected subgroup of 100 women from the MRU site (50 women from each contraceptive arm), blood and cervical cytobrushes were collected at trial initiation, prior to injection (D0), and 1 week after the 24‐week injection, that is, at 25 W (in total 3 injections for the DMPA‐IM group and 4 injections for the NET‐EN group). Several women were excluded due to pregnancy, HIV‐1 infection, or lost to follow‐up at 25 W, creating a modified intention‐to‐treat (mITT) group consisting of 93 women (DMPA‐IM *n* = 49, NET‐EN *n* = 44) (Figure 1). Serum levels of the study progestins (MPA and NET) and non‐study progestins (MPA, NET, etonogestrel, levonorgestrel, and gestodene) were determined at D0 and 25 W by ultra‐high performance liquid chromatography tandem mass spectrometry (UHPLC‐MS/MS) as previously described [40]. Based on the detection of non‐study progestin levels higher than 1.5 nM at either D0 or 25W [40], additional women were excluded, resulting in a per‐protocol (PP) group of 71 women (DMPA‐IM *n* = 41, NET‐EN *n* = 30) (Figure 1).

### 2.4 Sample Preparation and Flow Cytometry

PBMCs isolated from whole blood using standard Histopaque density centrifugation methods [46] and cervical cells collected from cytobrush samples as previously described [47] were frozen in liquid nitrogen at Clinical Research Labs in Durban. Cells were thawed and washed in PBS containing 1% charcoal‐stripped FCS (PBS‐FCS), and cervical cytobrush cells were filtered through 100 µm cell strainers, then counted using the TC20 automated cell counter (Bio‐Rad). One million PBMCs and the entire cervical cytobrush sample were stained for viability using Zombie Aqua (BV510, BioLegend 423102) for 15 min in the dark. Cells were washed in PBS‐FCS prior to staining with titrated antibodies in BD Brilliant Stain buffer (BD Bioscience 566349) for 20 min in the dark.

PBMC samples were stained at room temperature with CD3 AlexaFluor 700 (300324), CD4 APC Fire 750 (300560), and two different antibody combinations: Panel A: CCR5 BV421 (359118), CXCR4 BV605 (306522), CD69 PE‐Cy7 (310912), and α4β7 LPAM‐1 BV711 (BD Bioscience 740701); Panel B: CD38 BV605 (303532), CD25 PE‐Cy7 (356108), and HLA‐DR BV711 (307644).

Cervical cytobrush samples were stained on ice with CD3 FITC (300440), CD4 APC Fire 750 (300560), CCR5 APC (359122), CXCR4 BV605 (306522), CD25 PE (356104), CD69 PE‐Cy7 (310912), and α4β7 LPAM‐1 BV711 (BD Bioscience 740701). All antibodies were purchased from BioLegend, except for α4β7 LPAM‐1 BV711. Cells were washed in PBS‐FCS then fixed with 1 X BD CellFix (BD Bioscience 340181). Multiparameter flow cytometry was carried out using the Becton Dickinson LSRII and analyzed using FlowJo version 10.6 (Treestar Inc., Ashland, Ore). Fluorescence minus one controls were used to gate specific cell populations (Figures S1 and S2). Results are shown as frequency (percentage), expression (MFI) or cell numbers. Data for cell populations were excluded (serum: 25 W *n* = 1, cytobrush: D0 *n* = 19, and 25 W *n* = 13 excluded) if the number of viable CD3^+^ cells in that donor was <100, resulting in a difference in *n* values between serum and cytobrushes of reported data (Supporting Tables). All experiments were performed between August 2019 and January 2021. The researchers processing samples were blinded to the allocated contraceptive arms.

### 2.5 Statistical Analysis

Baseline characteristics were compared between arms using Pearson's Chi‐squared test or Fisher's exact test (Table 1). Age at baseline was compared between arms using a *t‐*test (Table 1). Statistical analysis was performed in R (version 4.2.2), using the tidyverse packages (version 1.3.2) for data manipulation. The Benjamini‐Hochberg method in the rstatix package (version 0.7.2) was used to obtain the false discovery rate (FDR) for multiple comparisons. Within‐group comparisons (D0 DMPA‐IM vs. 25 W DMPA‐IM or D0 NET‐EN vs. 25 W NET‐EN) were performed using Wilcoxon signed rank tests, and between‐group comparisons (D0 DMPA‐IM vs. D0 NET‐EN, 25 W DMPA‐IM vs. 25 W NET‐EN or change [25W‐D0] DMPA‐IM vs. change [25W‐D0] NET‐EN) were performed using the Wilcoxon rank sum test or Mann‐Whitney test. Heatmaps were drawn using GraphPad Prism (v9), indicating percentage change from baseline (change/D0) X 100). Statistical significance is indicated by a *p* value <0.05.

**TABLE 1:** Baseline characteristics of WHICH trial participants (mITT).

	Baseline (D0)				
	DMPA‐IM		NET‐EN		DMPA‐IM vs. NET‐EN
		*n*		*n*	*p* value^a^
**Age (years), mean (SD)**	25 (3.9)	49	24.8 (4.2)	44	0.85
**BMI, median (IQR)**	27.7 (24.5–32.8)	49	25.5 (23.4–29.3)	44	0.060
**BMI category, (%)**		49		44	0.25
Underweight	0 (0)		1 (2)		
Normal	16 (33)		19 (43)		
Overweight	14 (29)		14 (32)		
Obese	19 (39)		10 (23)		
**Ethnicity, (%)**		49		44	0.85
Xhosa	3 (6)		4 (9)		
Zulu	45 (92)		40 (91)		
Mixed race	0 (0)		0 (0)		
Other African ethnicity	1 (2)		0 (0)		
**Previous use of method** ^b^ **(%)**		49		44	
DMPA‐IM	39 (80)		28 (64)		0.087
NET‐EN	10 (20)		5 (11)		0.24
**Sexual dysfunction (ASEX), (%)**	3 (6)	49	4 (9)	44	0.59
**Depression (BDI), (%)**	1 (2)	49	0 (0)	44	0.34
**Feeling sad for no reason** ^c^ **(%)**	2 (4)	49	3 (7)	43	0.54
**No menstruation** ^c^ **(%)**	9 (18)	49	8 (19)	43	0.98
**Painless menstruation** ^c^ **(%)**	32 (80)	40	21 (60)	35	0.058
**No sexual intercourse** ^c^ **(%)**	5 (10)	49	12 (28)	43	**0.029**
**Never use a condom during intercourse** ^c^ **(%)**	6 (14)	44	4 (13)	31	0.93
**Decreased sexual desire** ^c^ **(%)**	2 (4)	49	5 (12)	43	0.17
**Marital status, (%)**		49		44	0.22
Single	49 (100)		42 (95)		
Married	0 (0)		2 (5)		

Abbreviations: ASEX, Arizona Sexual Experiences Scale [45]; BDI, Beck's Depression Inventory; BMI, body mass index.

^a^ *p* values are unadjusted.

^b^ Prior to exclusion period, numbers that responded (%).

^c^ Any occurrence in the last 3 months (%). Age compared using a two‐sample *t*‐test, all other characteristics compared using Pearson's Chi‐squared test or Fisher's exact test, where appropriate.

## 3 Results

### 3.1 Demographics

No differences in baseline characteristics between the two contraceptive arms were detected for the whole WHICH cohort (*n* = 521) [45], nor the population from whom samples were analyzed in this secondary study by flow cytometry (*n* = 93, Table 1), except for “no sexual intercourse” (Table 1) (mITT analysis).

### 3.2 Impact of DMPA‐IM and NET‐EN on Select Systemic CD4+ HIV‐1 Target Cells

**FIGURE 2:**
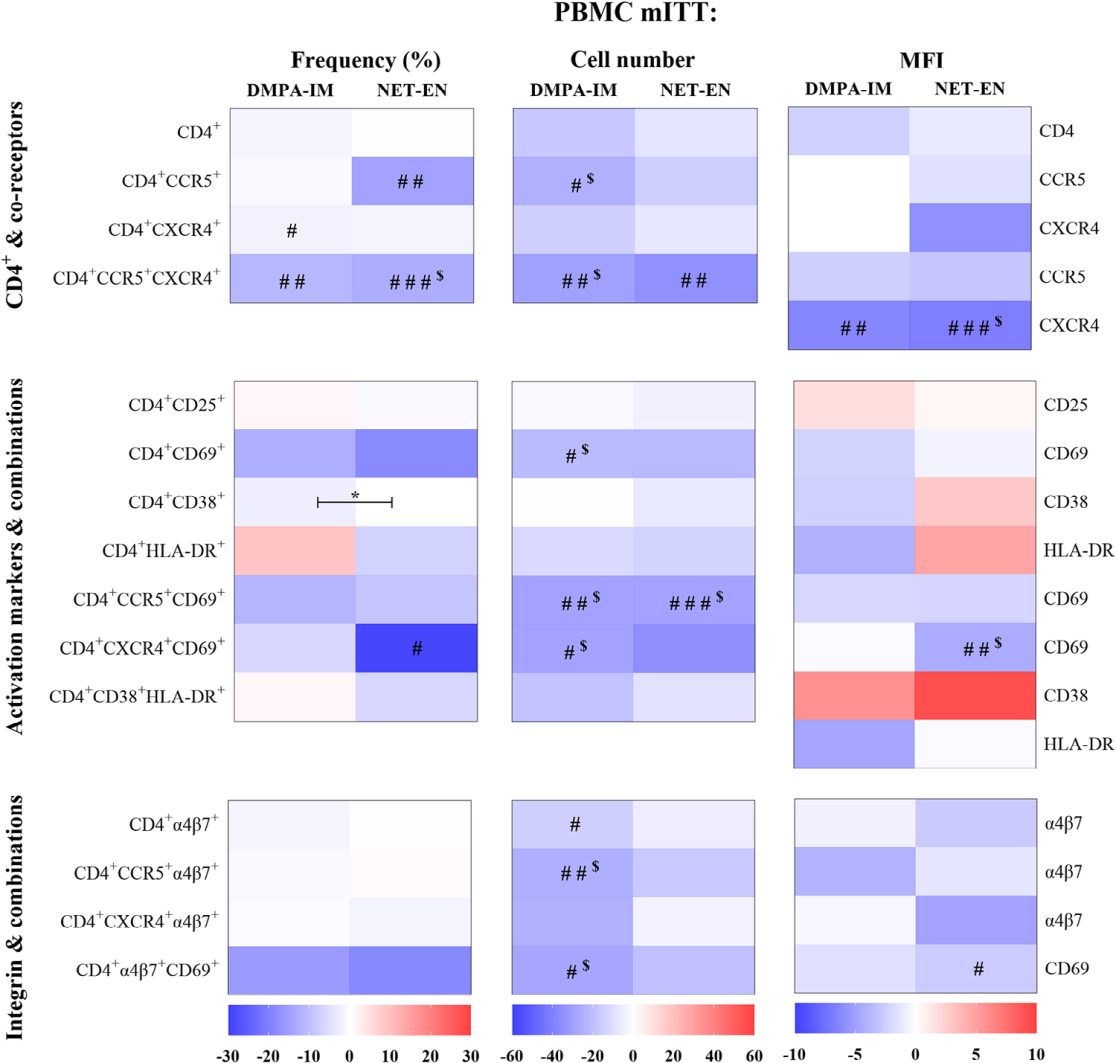
DMPA‐IM and NET‐EN similarly decrease the frequency and number of systemic CD4^+^ cells and expression of cell surface markers on most CD4^+^ cell populations investigated. Heatmaps show median percentage change from D0 to 25 W for frequency, cell number, and MFI of PBMC CD4^+^ cells (mITT) (Table S1). The marker analyzed for MFI is indicated on the right of that column. Statistical analysis was performed using raw data (Tables S2–S4); unadjusted *p* values are shown. Significance is indicated as # for within‐group (D0 to 25 W) or * for between‐group (DMPA‐IM vs. NET‐EN absolute change 25W‐D0) comparisons, where ### *p* < 0.001, ## *p* < 0.01, #/* *p* < 0.05. *n* = 49 DMPA‐IM (D0 & 25 W), *n* = 44 (D0), *n* = 43–44 (25 W) NET‐EN; ^$^ indicates the differences that remained statistically significant after adjusting for multiple comparisons (false discovery rate, FDR).

We evaluated the effects of DMPA‐IM and NET‐EN, alone or relative to each other, on 15 CD4^+^ cell populations in PBMCs collected at D0 and 25 W from women randomized to DMPA‐IM (*n* = 49) or NET‐EN (*n* = 44). The heatmap of the mITT analysis (Figure 2) reveals a clear trend whereby both contraceptives decreased the frequency (%) and cell numbers of most cell populations expressing HIV‐1 co‐receptors or activation markers and combinations thereof. A similar trend is evident for cell numbers to decrease for most cells expressing α4β7 alone or in combination with a co‐receptor and/or an activation marker (Figure 2, Tables S1–S4). Expression of co‐receptors and α4β7 on CD4^+^ cells also shows a trend to decrease with both contraceptives (Figure 2, Tables S1–S4). Although some of the apparent decreases within contraceptive arms were non‐significant, several were statistically significant for both contraceptives. This is particularly so for decreases in frequency of cells expressing co‐receptors (DMPA‐IM: CD4^+^CXCR4^+^ and CD4^+^CCR5^+^CXCR4^+^; NET‐EN: CD4^+^CCR5^+^ and CD4^+^CCR5^+^CXCR4^+^) and for cell numbers of most cell populations investigated for DMPA‐IM (CD4^+^CCR5^+^, CD4^+^CCR5^+^CXCR4^+^, CD4^+^CD69^+^, CD4^+^CCR5^+^CD69^+^, CD4^+^CXCR4^+^CD69^+^, CD4^+^α4β7^+^, CD4^+^CCR5^+^α4β7^+^, and CD4^+^α4β7^+^CD69^+^), and some for NET‐EN (CD4^+^CCR5^+^CXCR4^+^, and CD4^+^CCR5^+^CD69^+^). Of note, there is only one significant difference (frequency CD4^+^CD38^+^) and there are few apparent non‐significant differences between DMPA‐IM and NET‐EN in the effects investigated (Figure 2). After FDR analysis the between‐group significant difference in the mITT (+FDR) analysis for CD4^+^CD38^+^ cells was lost (Tables S1 and S2). Additionally, all within‐group statistically significant effects on frequency and expression were lost in the DMPA‐IM arm, while in the NET‐EN arm some remained (frequency CD4^+^CCR5^+^CXCR4^+^, expression of CXCR4 on CD4^+^CCR5^+^CXCR4^+^ and expression of CD69 on CD4^+^CXCR4^+^CD69^+^ cells) (Tables S2 and S3) in the FDR analysis. In contrast, 7/8 (CD4^+^CCR5^+^, CD4^+^CCR5^+^CXCR4^+^, CD4^+^CD69^+^, CD4^+^CCR5^+^CD69^+^, CD4^+^CXCR4^+^CD69^+^, CD4^+^CCR5^+^α4β7^+^, and CD4^+^α4β7^+^CD69^+^) and 1/2 (CD4^+^CCR5^+^CD69^+^cells) of the statistically significant within‐group decreases in cell numbers in the DMPA‐IM and NET‐EN arms, respectively, remained significant in the mITT (+FDR) analysis (Table S4). At D0, the frequency and number of total CD4^+^ cells in PBMCs were similar across study arms (Tables S2 and S4).

### 3.3 Impact of DMPA‐IM and NET‐EN on Select CD4+ HIV‐1 Target Cells in the FGT

**FIGURE 3:**
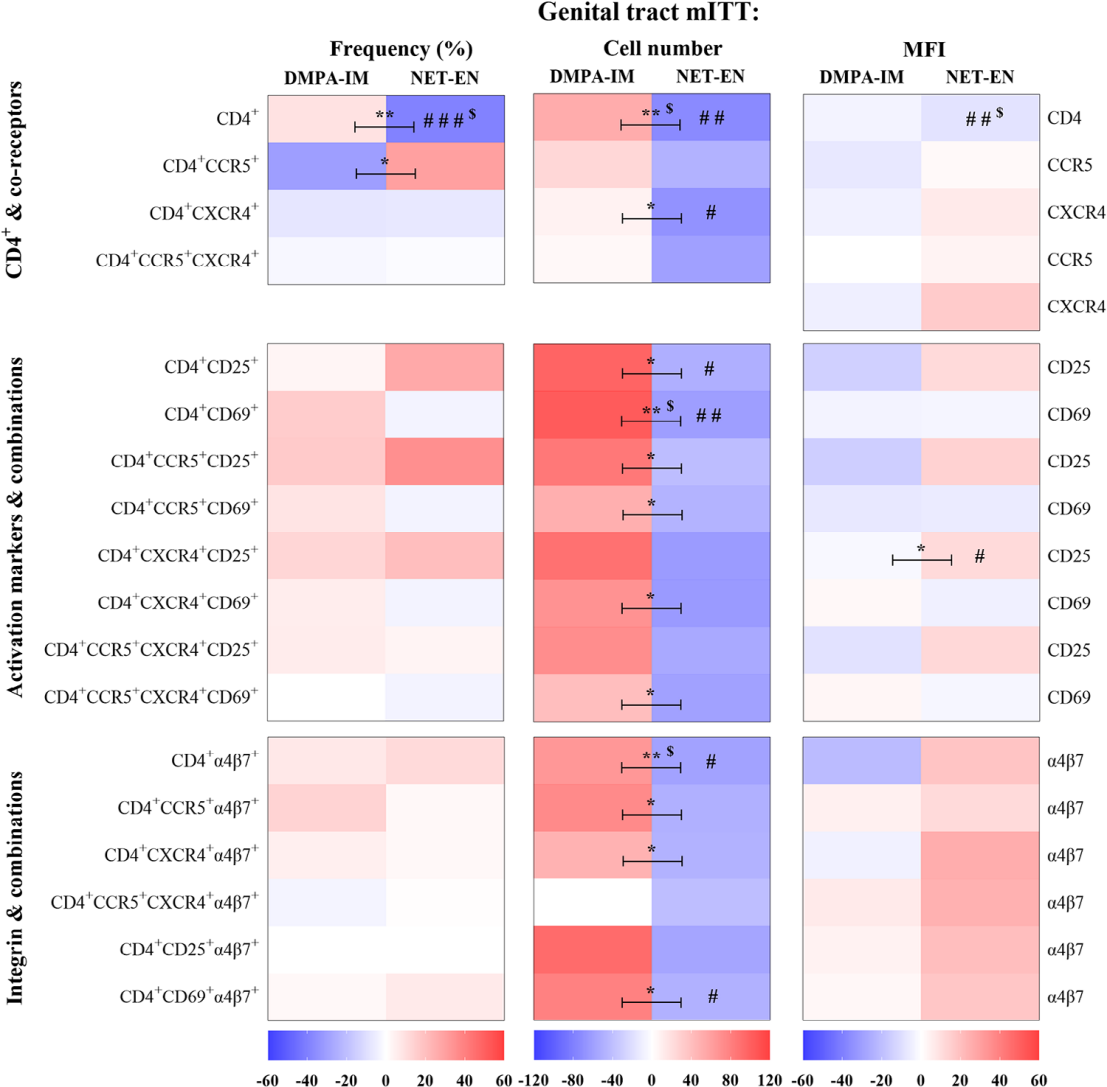
DMPA‐IM and NET‐EN have opposite effects on the expression of genital tract cell surface markers and numbers of CD4^+^ cell populations investigated. Heatmaps were generated and analyzed as described for Figure 2, except using FGT mITT data (Tables S5–S8): *n* = 35 (D0), *n* = 37 (25 W) DMPA‐IM, *n* = 39 (D0 & 25 W) NET‐EN; ^$^ indicates the differences that remained statistically significant after adjusting for multiple comparisons (false discovery rate, FDR).

We also evaluated the effects of DMPA‐IM and NET‐EN, alone or relative to each other, on 18 CD4^+^ cell populations from cytobrushes collected at D0 and 25 W from women randomized to DMPA‐IM (*n* = 35–37) or NET‐EN (*n* = 39). The mITT heatmap for the FGT (Figure 3) reveals contrasting results to those observed for PBMCs (Figure 2). Most noteworthy is the finding that the numbers of all the cervical CD4^+^ cell populations that express co‐receptors, activation markers or α4β7, or combinations thereof, tend to increase from D0 to 25 W in DMPA‐IM users but to decrease from D0 to 25 W in NET‐EN users (Figure 3, Tables S5–S8). A statistically significant difference was detected between DMPA‐IM and NET‐EN users for 12/18 of these cell populations (CD4^+^, CD4^+^CXCR4^+^, CD4^+^CD25^+^, CD4^+^CD69^+^, CD4^+^CCR5^+^CD25^+^, CD4^+^CCR5^+^CD69^+^, CD4^+^CXCR4^+^CD69^+^, CD4^+^CCR5^+^CXCR4^+^CD69^+^, CD4^+^α4β7^+^, CD4^+^CCR5^+^α4β7^+^, CD4^+^CXCR4^+^α4β7^+^, and CD4^+^CD69^+^α4β7^+^), with cell numbers being greater for DMPA‐IM. A few within‐contraceptive arm effects were significant for individual cell populations for NET‐EN (CD4^+^, CD4^+^CXCR4^+^, CD4^+^CD25^+^, CD4^+^CD69^+^, CD4^+^α4β7^+^, and CD4^+^CD69^+^α4β7^+^) but not DMPA‐IM. Interestingly, the frequency of total CD4^+^ cells was significantly different between contraceptives, with a non‐significant increase for DMPA‐IM and a significant decrease detected for NET‐EN users. There appeared to be a weak trend for frequency of cervical CD4^+^ cells expressing activation and integrin markers to mostly increase from D0 to 25 W, in both contraceptive arms, but more so for DMPA‐IM than NET‐EN. There also appeared to be a weak trend for frequency of most cervical CD4^+^ cells expressing only co‐receptors to decrease from D0 to 25 W in both contraceptive arms, except for CD4^+^CCR5^+^ cell frequency which tended to increase in the NET‐EN group. However, none of these frequency trends was significant for individual populations within arms, while relative frequency effects for only CD4^+^CCR5^+^ cells were significant between arms. There also appeared to be weak trends for expression of most cell surface markers to decrease for DMPA‐IM but to increase for NET‐EN, but only two cell populations within arms (NET‐EN: expression of CD4 on CD4^+^ cells and expression of CD25 on CD4^+^CXCR4^+^CD25^+^ cells) and one cell population between arms (expression of CD25 on CD4^+^CXCR4^+^CD25^+^ cells) showed significant effects. FDR adjustments resulted in loss of most within‐ and between‐group significant differences in the mITT (+FDR) analysis, with only a few significant effects being retained, including for decreased CD4^+^ frequency and expression of CD4^+^ on CD4^+^ cells for NET‐EN and between‐group differences in cell numbers of CD4^+^, CD4^+^CD69^+^, and CD4^+^αbβ7^+^ cells (Tables S5–S8). The frequency and number of cervical CD4^+^ cells at baseline were similar across study arms (Tables S6 and S8).

### 3.4 Impact of Non‐Study Progestin Use on Select CD4+ Cell Populations

To assess the potential impact of non‐study progestin use on our mITT data, some participants were excluded (see Methods), and a PP analysis was performed for both the PBMC (DMPA‐IM *n* = 41, NET‐EN *n* = 29–30) and cytobrush samples (DMPA‐IM *n* = 29–30, NET‐EN *n* = 26). No statistically significant differences between contraceptive arms were detected in baseline demographic characteristics in the PP analysis (Table S9).

**FIGURE 4:**
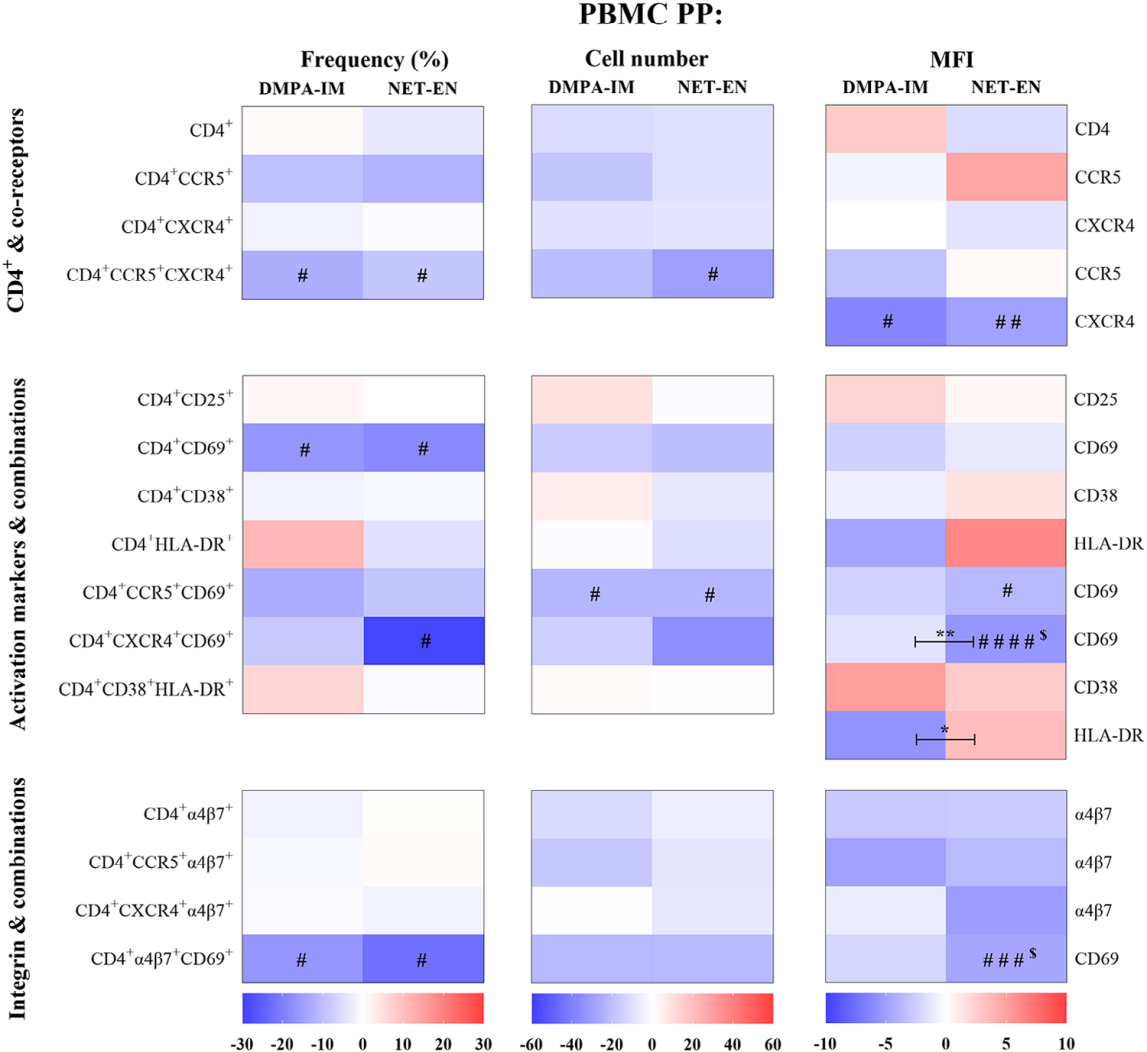
DMPA‐IM and NET‐EN similarly decrease systemic frequency and number of CD4^+^ cells and expression of cell surface markers on systemic CD4^+^ cells investigated after correction for non‐study progestin. Heatmaps were generated and analyzed as described for Figure 2, except using PBMC PP data (Tables S10–S13): *n* = 41 DMPA‐IM (D0 & 25 W), *n* = 30 (D0), *n* = 29–30 (25 W) NET‐EN; ^$^ indicates the differences that remained statistically significant after adjusting for multiple comparisons (false discovery rate, FDR).

PP analysis of the PBMC data (Figure 4, Tables S10–S13) showed the same trends of decreasing frequency and numbers of select HIV‐1 target cell populations and expression of cell surface markers as for the mITT analysis for most cell populations investigated (Figure 2). However, about 25% of the significant within‐contraceptive arm decreases in the mITT analysis were not detected in the PP analysis. Significant differences between contraceptives were detected for 2/15 cell populations in the PP analysis (expression of CD69 and HLA‐DR on CD4^+^CXCR4^+^CD69^+^ and CD4^+^CD38^+^HLA‐DR^+^ cells, respectively), compared to 1/15 in the mITT analysis. All but two of the significant differences (expression of CD69 on CD4^+^CXCR4^+^CD69^+^ and CD4^+^α4β7^+^CD69^+^ cells), in the PBMC PP analysis were lost in the PP (+FDR) analysis.

**FIGURE 5:**
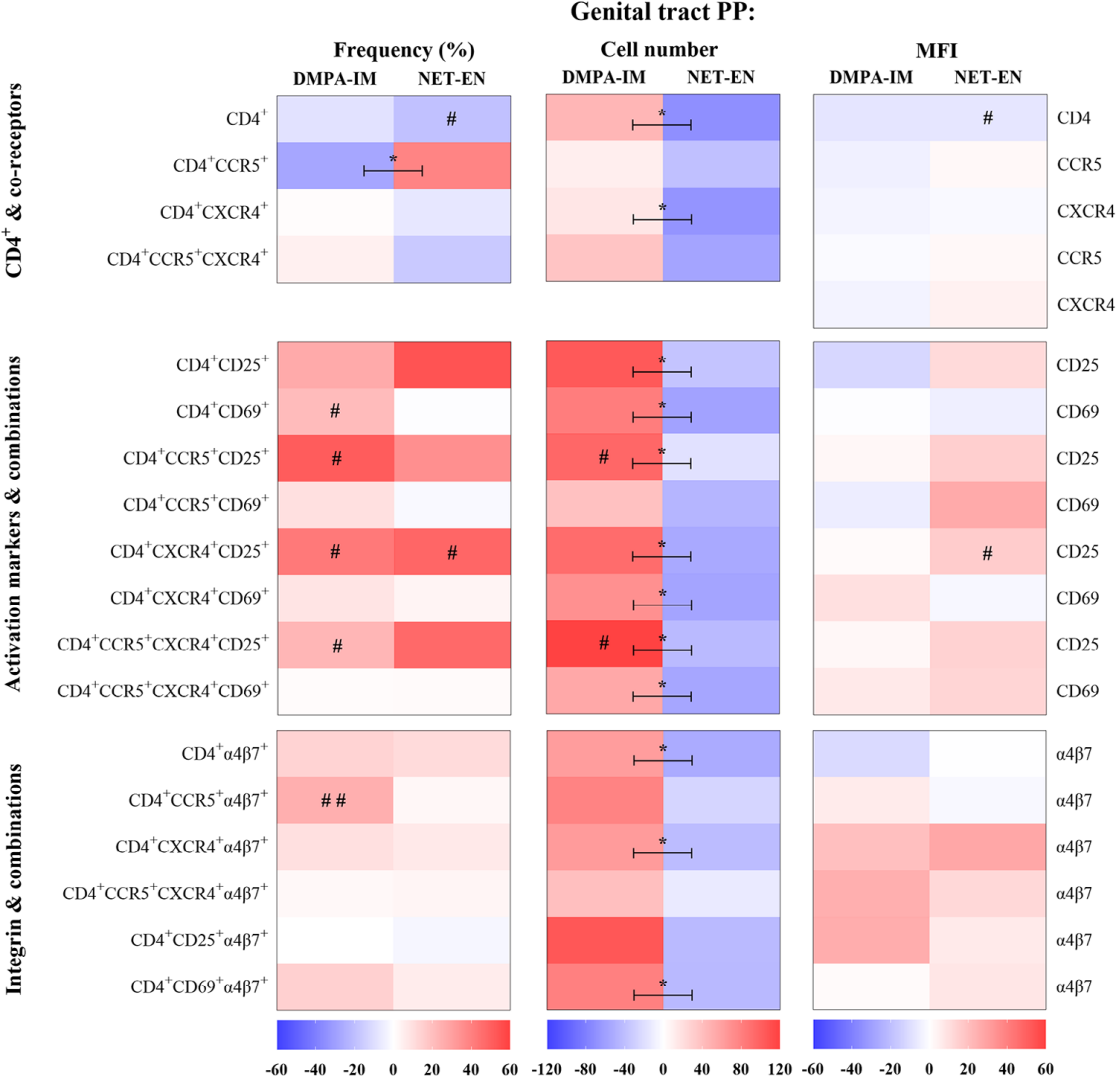
DMPA‐IM and NET‐EN differentially regulate the investigated genital tract CD4^+^ cell population numbers after correction for non‐study progestin. Heatmaps were generated and analyzed as described for Figure 2, except using FGT PP data (Tables S14–S17): *n* = 30 (D0), *n* = 29 (25 W) DMPA‐IM, *n* = 26 (D0 & 25 W) NET‐EN.

PP analysis of the FGT data (Figure 5, Tables S14–S17) showed the same trends as the mITT analysis (Figure 3). Most noteworthy is that in FGT HIV‐1 target cells investigated, all 12/18 statistically significant differences between DMPA‐IM and NET‐EN in cell numbers were detected in both the PP and mITT analyses (CD4^+^, CD4^+^CXCR4^+^, CD4^+^CD25^+^, CD4^+^CD69^+^, CD4^+^CCR5^+^CD25^+^, CD4^+^CXCR4^+^CD25^+^, CD4^+^CXCR4^+^CD69^+^, CD4^+^CCR5^+^CXCR4^+^CD25^+^, CD4^+^CCR5^+^CXCR4^+^CD69^+^, CD4^+^α4β7^+^, CD4^+^CXCR4^+^α4β7^+^, and CD4^+^CD69^+^α4β7^+^), with a gain in statistical significance for increased CD4^+^CCR5^+^CD25^+^ and CD4^+^ CCR5^+^CXCR4^+^CD25^+^ cell numbers within the DMPA‐IM arm. In contrast to the PBMC PP results, the FGT PP adjustment showed significant increases in frequency for CD4^+^CD69^+^, CD4^+^CCR5^+^CD25^+^, CD4^+^CXCR4^+^CD25^+^, CD4^+^CCR5^+^CXCR4^+^CD25^+^, and CD4^+^CCR5^+^α4β7^+^ cell populations for women on DMPA‐IM. In the NET‐EN arm, a statistically significant increased frequency of CD4^+^CXCR4^+^CD25^+^ cells was detected in the PP but not the mITT analysis. All the significant effects in the FGT PP analysis were lost in the PP (+FDR) adjustment, although the between‐group *p* values were near‐significant (*p* = 0.062–0.065) (Table S17).

## 4 Discussion

We provide robust data on the individual as well as relative effects of DMPA‐IM and NET‐EN on select CD4^+^ HIV‐1 target cells in the blood and FGT, to provide insight into likely effects of these contraceptives on HIV‐1 acquisition. This is the first randomized head‐to‐head trial to investigate the impact of DMPA‐IM and NET‐EN on HIV‐1 target cells. Additionally, it is the first randomized study to do this concurrently in the blood and FGT, at peak progestin levels 1 week after 3 or 4 injections of DMPA‐IM or NET‐EN, respectively. We present results from both without (mITT) and with (PP) correcting for non‐study progestins, as well as without and with multiple comparison adjustments for both. We considered both visible heatmap trends for functional categories of immune cells, as well as *p* values where *p* < 0.05 indicates statistical significance. We have reported all comparisons but interpreted the data without FDR adjustments, given that all systemic and genital tract immune cell biomarkers were selected based on the literature, and all planned comparisons were specified a priori rather than determined post hoc [48, 49, 50].

We show in our mITT analysis that both DMPA‐IM and NET‐EN use (Figure 2) tend to decrease the frequency and numbers of systemic CD4^+^ cells expressing the HIV‐1 co‐receptors CCR5 and CXCR4, the activation marker CD69 as well as the integrin α4β7, alone or in combination with each other. Of note, the relative effects of DMPA‐IM and NET‐EN are very similar, with only the relative frequency of CD4^+^CD38^+^ cells differing significantly between contraceptive groups. The systemic PP results obtained after correction for non‐study progestins (Figure 4) are very similar to the mITT results, while FDR adjustment resulted in the loss of most statistically significant within‐ and between‐group comparisons. Consistent results between systemic mITT and PP analyses suggest that the findings are robust and not significantly affected by imperfections in the study population and are not related to false discovery.

The observation that the results are comparable after correction for non‐study progestins suggests that the systemic mITT findings are not substantially confounded by non‐study progestins. Although only a few significant effects were detected, both analyses do, however, suggest a similar trend to decrease systemic susceptibility to HIV‐1 infection by decreasing the frequency and number of some HIV‐1 target cell populations and the MFIs of related cell surface markers. This appears, in particular, to be for effects on CD4^+^ cells expressing α4β7 and CD69, which are reportedly highly permissive to HIV‐1 infection [12]. Taken together, our results suggest that DMPA‐IM and NET‐EN are unlikely to increase systemic HIV‐1 infectivity at 25 W after initiation, due to decreases in select HIV‐1 target cell populations.

Our systemic PP results showing few effects on CD4^+^ cell numbers are consistent with those reported for DMPA‐IM or NET‐EN from a non‐randomized study sampling at nadir progestin levels (90 and 180 days post‐initiation), although this study did not report on frequency or MFI for CD4^+^, CD4^+^CCR5^+^, and CD4^+^CD69^+^ cells [34]. Achilles et al. did not statistically evaluate effects between the DMPA‐IM and NET‐EN arms [34], whereas we report very few statistically significant differences between these contraceptives. Our systemic results are also consistent with another study that did not detect significant effects of injectable contraceptive use on CD4^+^CCR5^+^ cell frequency compared to women using no long‐term contraception [26]. Several other non‐randomized studies investigating systemic CD4^+^ cells reported increases or decreases that mostly appear inconsistent with our data for DMPA‐IM [29, 30]. One study [14] reported increased frequency of CD4^+^α4β7^+^ cells in women on DMPA‐IM 1 month after injection, unlike in our study, but no effect on CD4^+^CCR5^+^ cell frequency or MFI, consistent with our study. Previous ex vivo data from our laboratory offer possible insights into the relationships between changes in systemic CD4^+^ immune cell populations, progestin levels, and HIV‐1 infectivity. We previously reported that 100 nM MPA, but not NET, increased expression of CD69, CD25, and CCR5 on CD4^+^ cells in PBMCs ex vivo [22]. Dose‐response data further showed that MPA significantly increased HIV‐1 infection of PBMCs at 50 nM but not 10 nM MPA [22]. Considered together with our clinical data, these results suggest that concentrations of MPA greater than 10 nM may be required in vivo to obtain the effects on systemic cells observed ex vivo, consistent with our finding for median peak MPA levels in the WHICH trial cohort of 6.59 nM [40].

Our FGT results contrast with our systemic results, with DMPA‐IM and NET‐EN exhibiting several significantly different effects on immune cells compared to each other, in particular regarding cell numbers. Taken together the FGT findings show that DMPA‐IM increases the frequency and cell numbers of several HIV‐1 target cell populations investigated, while NET‐EN tends to decrease these populations, suggesting that DMPA‐IM may increase while NET‐EN may decrease the vulnerability to HIV‐1 acquisition 25 W after initiation. Interesting trends of increased FGT CD4^+^ cell numbers but lower MFI for many of these cell populations were observed in the DMPA‐IM arm in the mITT population. This apparent discordance was also evident for the NET‐EN arm, but in the opposite direction. The implications are unclear, since they suggest opposite effects on HIV‐1 susceptibility. However, in the PP population, significant increases in numbers of CD4^+^CCR5^+^CD25^+^ and CD4^+^CCR5^+^CXCR4^+^CD25^+^ cells, with no trend to decrease the MFI for these markers, could support an increase in HIV‐1 susceptibility in the FGT for the DMPA‐IM arm. Not only were more significant increases from D0 to 25 W in frequency and cell numbers detected in the DMPA‐IM arm, but all the significant between‐group differences in cell numbers were retained in the PP analysis. This suggests that the presence of non‐study progestins confounds the FGT mITT results [40]. The PP data are likely more robust and less variable than the mITT data, due to fewer and/or lower concentrations of non‐study progestins with different effects compared to the study progestin, in the serum of that population. The systemic mITT data appeared less confounded by non‐study progestins, suggesting that FGT immune cells are more sensitive to progestin levels than systemic immune cells. We speculate that this may be due to relatively higher concentrations of progesterone and androgen steroid receptors in the FGT cells compared to immune cells in the blood, which could increase the sensitivity to progestins. It is possible that differences in numbers of women reporting no sexual intercourse prior to initiation could have contributed to the differences between contraceptive groups in frequency and numbers of FGT CD4^+^ cell populations [51] in the mITT population of women. However, this is unlikely since these CD4^+^ cell changes are even more pronounced in the PP population, where no such significant difference in sexual behavior was detected between contraceptive groups (Table S9). The significant increase in frequency of FGT CD4^+^ cells expressing combinations of HIV‐1 co‐receptors together with the CD69 or CD25 activation markers and CD4^+^CCR5^+^α4β7^+^ cells in the PP analysis, supports an increase in HIV‐1 susceptibility in the FGT for DMPA‐IM users. The numbers of FGT CD4^+^ cells expressing the mucosal homing receptor α4β7 alone or in combination with CD69 were reduced in NET‐EN users in the mITT analysis, supporting a decrease in HIV‐1 susceptibility in the FGT for NET‐EN users, although this effect was not significant in the PP analysis. Besides serving as a marker for immune activation, CD69 is also a marker for tissue retention [52]. The majority of CD4^+^ cells productively infected by HIV‐1 in vitro have been shown to express CD69 or CD25 [16, 53]. Our results on CD4^+^ populations expressing CD69, CD25, and α4β7 suggest that DMPA‐IM may increase the ability of the FGT to retain highly permissive HIV‐1 target cells and support productive HIV‐1 infection, while NET‐EN may reduce HIV‐1 infection 25 W after initiation.

Our FGT results for DMPA‐IM are consistent with those from a substudy of the ECHO trial, where no significant effects were detected for frequency of CD4^+^, CD4^+^CCR5^+^, and CD4^+^α4β7^+^ cells [24], but not with our PP analysis data showing increased frequency of CD4^+^CCR5^+^α4β7^+^ cells in the DMPA‐IM arm. However, these authors did not investigate effects on CD4^+^ populations expressing CD69, CXCR4, and CD25, or combinations thereof, many of which we found increased in frequency and/or cell numbers in the mITT analysis for the DMPA‐IM arm. Although we did not investigate CD4^+^HLA‐DR^+^ cells, the observed trend of increased FGT cellular frequencies and numbers of CD4^+^ cells expressing activation markers is consistent with increased activation of CD4^+^ HIV‐1 target cells in the FGT of DMPA‐IM users. Our FGT results for NET‐EN are consistent with a non‐randomized study, which did not detect a change in frequency of CD4^+^CCR5^+^ cells, but inconsistent with their results for an unchanged frequency of CD4^+^ cells [34], for which we detected a decreased frequency. Although a parallel study, their study only determined individual effects of DMPA‐IM and NET‐EN in the FGT and investigated fewer populations compared to our study [34].

Our published ex vivo data showing a significant decrease in HIV‐1 infection with 100 nM NET in cervical explants [23] are consistent with the possible protective effects of NET‐EN suggested by our current FGT clinical data, although the median peak serum levels of NET in the WHICH cohort are lower at 13.6 nM [40]. We also reported previously that 10 nM MPA increases R5 HIV‐1 infection in endocervical but not ectocervical tissue explants ex vivo, while 100 nM MPA does so in both tissues [23]. Our clinical data suggesting increased HIV‐1 cell numbers of select FGT HIV‐1 target cells are consistent with the ex vivo endocervix data, since cytobrush sampling contains endocervical cells [54]. Overall, our FGT data suggest possible biological mechanisms whereby DMPA‐IM, but not NET‐EN, may enhance susceptibility to HIV‐1 infection, and they suggest that NET‐EN may be a safer alternative for women vulnerable to HIV‐1 infection.

It is not surprising that our results differ in some respects from published data, given the multiple potential sources of differences in these studies. These include differences in demographic/intrinsic factors such as age, ethnicity and body mass index, different numbers of injections, potential levels of non‐study progestins, sample size, sampling methods and times, flow cytometry markers investigated, and methods of data analysis. Comparisons between contraceptive arms in clinical trials that are not randomized are likely confounded by several of the above factors. No other studies have reported on the effects at peak progestin levels after several injections of both contraceptives. Some studies reported sampling 1‐month post‐injection, or prior to the next injection when progestin levels would be at their lowest concentration, or after only a single injection [24, 28, 34]. After only one DMPA‐IM injection, variable effects are reported regarding bleeding patterns, ovulation, and hormonal effects [55, 56, 57], which become more consistent after two injections and are likely to affect immune cell markers in the FGT. We and others [10, 34, 39, 43, 44, 58–61] have previously reported that incorrect self‐reporting of non‐study progestin use is widespread, particularly for DMPA‐IM as the major contraceptive used in the study site area [40], likely blunting reported DMPA‐IM effects. Only one other flow cytometry study on these contraceptives has excluded participants for objectively quantified non‐study progestin use [34]. Some studies report sample numbers similar but slightly lower than in our study [13, 14, 25, 27, 28, 30, 32–34], while others were substantially lower and/or uneven [24, 29, 31, 36, 62]. Most of the significant effects detected in our study were for cell numbers, whereas limited previous contraceptive studies also reported cell numbers [34, 35]. Most reported flow cytometry data are not adjusted for potential FDR [14, 26, 29–31, 33, 62, 63] while some are [24, 28, 34, 36]. FDR analysis may be too stringent to detect biologically relevant changes in small sample sizes when targeting specific cell populations [64, 65, 66].

As with all studies, ours had some limitations. Although most studies do not report cell numbers, some of ours were low relative to some reports [16, 34]. We did not investigate the effect of sexually transmitted infections or the cervicovaginal microbiome in our cohort on immune cell changes in the FGT [67, 68]. We also did not investigate all possible cell‐surface markers, including FGT markers for Th17 cells or CD38 to enable direct comparisons of these populations with other studies [24, 28]. Since many women in the WHICH cohort were overweight or obese (23%–39%) and mostly Zulu (>90%), our data do not exclude the possibility that women with lower weight, or from different ethnic groups, with higher MPA serum levels, may exhibit more changes in HIV‐1 target cell populations investigated. Additionally, since we did not record the participants’ menstrual cycle phases at baseline, possible differences in phases between contraceptive arms could have confounded the differences detected between immune cell populations investigated.

In conclusion, we report a robust comparison of systemic and FGT CD4^+^ cellular markers of HIV‐1 susceptibility in a cohort of women randomized to DMPA‐IM and NET‐EN, at peak progestin serum levels after the 6‐month injection. Results with and without correction for misreporting of non‐study progestin use suggest that selected FGT HIV‐1 target CD4^+^ cell populations are more sensitive to confounding effects of non‐study progestin use than systemic CD4^+^ HIV‐1 target cell populations. Our results suggest that DMPA‐IM and NET‐EN use have minimal and similar effects on select systemic HIV‐1 target cells and may even reduce systemic HIV‐1 infection susceptibility at 25 W after initiation. In contrast, our FGT results suggest that DMPA‐IM may enhance the susceptibility to HIV‐1 acquisition compared to NET‐EN due to an increase in select HIV‐1 target CD4^+^ cell population numbers and frequencies. Our results further suggest that DMPA‐IM may increase the ability of the FGT to retain some highly permissive CD4^+^ HIV‐1 target cells recruited from the blood.

## Disclosure

The funders had no role in study design, data collection and analysis, decision to publish, or preparation of the manuscript.

## Ethics Statement

The authors confirm that the ethical policies of the journal, as noted on the journal's author guidelines page, have been adhered to, and the appropriate ethical review committee approval has been received. The study was approved by the University of Witwatersrand Faculty of Health Sciences Human Research (M180528), East London Hospital Institutional ethics committees, and Human Research Ethics Committee at the University of Cape Town (HREC 664/2018).

## Conflicts of Interest

The authors declare no conflicts of interest.

## Supporting information



Supporting Information

Supporting Information

## Data Availability

The data that supports the findings of this study are available in the supporting material of this article.

## References

[1] The Path That Ends AIDS: UNAIDS Global AIDS Update 2023 . Geneva: Joint United Nations Programme on HIV/AIDS; 2023. Licence: CC BY‐NC‐SA 3.0 IGO.

[2] United Nations Department of Economic and Social Affairs, Population Division (2022) . World Family Planning 2022: Meeting the Changing Needs for Family Planning: Contraceptive Use by Age and Method. UN DESA/POP/2022/TR/NO. 4, https://www.un.org/development/desa/pd/sites/www.un.org.development.desa.pd/files/files/documents/2023/Feb/undesa_pd_2022_world-family-planning.pdf.

[3] National Department of Health (NDoH), Statistics South Africa (Stats SA), South African Medical Research Council (SAMRC), ICF . South Africa Demographic and Health Survey 2016. Pretoria, South Africa and Rockville, Maryland, USA: NDoH, Stats SA, SAMRC, and ICF; 2019, http://dhsprogram.com/pubs/pdf/FR337/FR337.pdf.

[4] C. S. Morrison , P.‐L. Chen , C. Kwok , et al., “Hormonal Contraception and the Risk of HIV Acquisition: An Individual Participant Data Meta‐Analysis,” PLOS Medicine 12, no. 1 (2015): e1001778. 25612136 10.1371/journal.pmed.1001778 PMC4303292

[5] L. M. Noguchi , B. A. Richardson , J. M. Baeten , et al., “Risk of HIV‐1 Acquisition Among Women Who Use Different Types of Injectable Progestin Contraception in South Africa: A Prospective Cohort Study,” Lancet HIV 2, no. 7 (2015): e279–e287. 26155597 10.1016/S2352-3018(15)00058-2 PMC4491329

[6] J. Brind , S. J. Condly , S. W. Mosher , A. R. Morse , and J. Kimball , “Risk of HIV Infection in Depot‐Medroxyprogesterone Acetate (DMPA) Users: A Systematic Review and Meta‐analysis,” Issues in Law & Medicine 30, no. 2 (2015): 129–139. 26710371

[7] C. B. Polis , K. M. Curtis , P. C. Hannaford , et al., “An Updated Systematic Review of Epidemiological Evidence on Hormonal Contraceptive Methods and HIV Acquisition in Women,” Aids 30, no. 17 (2016): 2665–2683. 27500670 10.1097/QAD.0000000000001228 PMC5106090

[8] C. B. Polis , S. J. Phillips , K. M. Curtis , et al., “Hormonal Contraceptive Methods and Risk of HIV Acquisition in Women: A Systematic Review of Epidemiological Evidence,” Contraception 90, no. 4 (2014): 360–390. 25183264 10.1016/j.contraception.2014.07.009

[9] L. J. Ralph , S. I. McCoy , K. Shiu , and N. S. Padian , “Hormonal Contraceptive Use and Women's Risk of HIV Acquisition: A Meta‐Analysis of Observational Studies,” Lancet Infectious Diseases 15, no. 2 (2015): 181–189. 25578825 10.1016/S1473-3099(14)71052-7 PMC4526270

[10] K. Ahmed , J. M. Baeten , M. Beksinska , et al., “HIV Incidence Among Women Using Intramuscular Depot Medroxyprogesterone Acetate, a Copper Intrauterine Device, or a Levonorgestrel Implant for Contraception: A Randomised, Multicentre, Open‐Label Trial,” Lancet 394, no. 10195 (2019): 303–313. 31204114 10.1016/S0140-6736(19)31288-7 PMC6675739

[11] S. Dabee , C. Balle , M. Onono , et al., “Update on the Impact of Depot Medroxyprogesterone Acetate on Vaginal Mucosal Endpoints and Relevance to Sexually Transmitted Infections,” Current HIV/AIDS Reports 20, no. 4 (2023): 251–260. 37341916 10.1007/s11904-023-00662-0 PMC10403392

[12] V. R. Joag , L. R. McKinnon , J. Liu , et al., “Identification of Preferential CD4+ T‐Cell Targets for HIV Infection in the Cervix,” Mucosal Immunology 9, no. 1 (2016): 1–12. 25872482 10.1038/mi.2015.28

[13] G. Sciaranghella , C. Wang , H. Hu , et al., “CCR5 Expression Levels in HIV‐Uninfected Women Receiving Hormonal Contraception,” Journal of Infectious Diseases 212, no. 9 (2015): 1397–1401. 25895986 10.1093/infdis/jiv233 PMC4601918

[14] C. Tasker , A. Davidow , N. E. Roche , and T. L. Chang , “Depot Medroxyprogesterone Acetate Administration Alters Immune Markers for HIV Preference and Increases Susceptibility of Peripheral CD4(+) T Cells to HIV Infection,” Immunohorizons 1, no. 9 (2017): 223–235. 29188238 10.4049/immunohorizons.1700047 PMC5703073

[15] R. T. Trifonova , J. Lieberman , and D. van Baarle , “Distribution of Immune Cells in the human Cervix and Implications for HIV Transmission,” American Journal of Reproductive Immunology 71, no. 3 (2014): 252–264. 24410939 10.1111/aji.12198 PMC3943534

[16] C. M. Card , W. J. Rutherford , S. Ramdahin , et al., “Reduced Cellular Susceptibility to in Vitro HIV Infection Is Associated With CD4+ T Cell Quiescence,” PLoS ONE 7, no. 9 (2012): e45911. 23029309 10.1371/journal.pone.0045911 PMC3448692

[17] L. R. McKinnon , B. Nyanga , D. Chege , et al., “Characterization of a Human Cervical CD4+ T Cell Subset Coexpressing Multiple Markers of HIV Susceptibility,” Journal of Immunology 187, no. 11 (2011): 6032–6042. 10.4049/jimmunol.1101836 22048765

[18] A. L. Meditz , M. K. Haas , J. M. Folkvord , et al., “HLA‐DR+ CD38+ CD4+ T Lymphocytes Have Elevated CCR5 Expression and Produce the Majority of R5‐tropic HIV‐1 RNA in Vivo,” Journal of Virology 85, no. 19 (2011): 10189–10200. 21813616 10.1128/JVI.02529-10 PMC3196402

[19] J. Arthos , C. Cicala , E. Martinelli , et al., “HIV‐1 Envelope Protein Binds to and Signals Through Integrin alpha4beta7, the Gut Mucosal Homing Receptor for Peripheral T Cells,” Nature Immunology 9, no. 3 (2008): 301–309. 18264102 10.1038/ni1566

[20] C. Cicala , J. Arthos , and A. S. Fauci , “HIV‐1 Envelope, Integrins and Co‐Receptor Use in Mucosal Transmission of HIV,” Journal of Translational Medicine 9, no. 1 (2011): S2. 21284901 10.1186/1479-5876-9-S1-S2 PMC3105502

[21] C. Cicala , E. Martinelli , J. P. McNally , et al., “The Integrin alpha4beta7 Forms a Complex With Cell‐Surface CD4 and Defines a T‐Cell Subset That Is Highly Susceptible to Infection by HIV‐1,” PNAS 106, no. 49 (2009): 20877–20882. 19933330 10.1073/pnas.0911796106 PMC2780317

[22] M. F. Maritz , R. M. Ray , A. J. Bick , et al., “Medroxyprogesterone Acetate, Unlike Norethisterone, Increases HIV‐1 Replication in Human Peripheral Blood Mononuclear Cells and an Indicator Cell Line, via Mechanisms Involving the Glucocorticoid Receptor, Increased CD4/CD8 Ratios and CCR5 Levels,” PLoS ONE 13, no. 4 (2018): e0196043. 29698514 10.1371/journal.pone.0196043 PMC5919616

[23] R. M. Ray , M. F. Maritz , C. Avenant , et al., “The Contraceptive Medroxyprogesterone Acetate, Unlike Norethisterone, Directly Increases R5 HIV‐1 Infection in human Cervical Explant Tissue at Physiologically Relevant Concentrations,” Scientific Reports 9, no. 1 (2019): 4334. 30867477 10.1038/s41598-019-40756-7 PMC6416361

[24] R. Bunjun , T. F. Ramla , S. Z. Jaumdally , et al., “Initiating Intramuscular Depot Medroxyprogesterone Acetate Increases Frequencies of Th17‐Like Human Immunodeficiency Virus Target Cells in the Genital Tract of Women in South Africa: A Randomized Trial,” Clinical Infectious Diseases 75, no. 11 (2022): 2000–2011. 35941737 10.1093/cid/ciac284 PMC9710690

[25] F. Bradley , M. Franzen Boger , V. Kaldhusdal , A. Ahlberg , G. Edfeldt , J. Lajoie , et al., “Multi‐Omics Analysis of the Cervical Epithelial Integrity of Women Using Depot Medroxyprogesterone Acetate,” PLOS Pathogens 18, no. 5 (2022): e1010494. 35533147 10.1371/journal.ppat.1010494 PMC9119532

[26] E. H. Byrne , M. N. Anahtar , K. E. Cohen , et al., “Association Between Injectable Progestin‐Only Contraceptives and HIV Acquisition and HIV Target Cell Frequency in the Female Genital Tract in South African Women: A Prospective Cohort Study,” Lancet Infectious Diseases 16, no. 4 (2016): 441–448. 26723758 10.1016/S1473-3099(15)00429-6 PMC5294917

[27] G. Edfeldt , J. Lajoie , M. Rohl , et al., “Regular Use of Depot Medroxyprogesterone Acetate Causes Thinning of the Superficial Lining and Apical Distribution of Human Immunodeficiency Virus Target Cells in the Human Ectocervix,” Journal of Infectious Diseases 225, no. 7 (2022): 1151–1161. 32780807 10.1093/infdis/jiaa514 PMC8974825

[28] I. N. Konstantinus , C. Balle , S. Z. Jaumdally , et al., “Impact of Hormonal Contraceptives on Cervical T‐Helper 17 Phenotype and Function in Adolescents: Results From a Randomized, Crossover Study Comparing Long‐Acting Injectable Norethisterone Oenanthate (NET‐EN), Combined Oral Contraceptive Pills, and Combined Contraceptive Vaginal Rings,” Clinical Infectious Diseases 71, no. 7 (2020): e76–e87. 31675420 10.1093/cid/ciz1063 PMC7755094

[29] J. Lajoie , A. Tjernlund , K. Omollo , et al., “Increased Cervical CD4(+)CCR5(+) T Cells among Kenyan Sex Working Women Using Depot Medroxyprogesterone Acetate,” Aids Research and Human Retroviruses 35, no. 3 (2019): 236–246. 30585733 10.1089/aid.2018.0188 PMC6434599

[30] K. Omollo , J. Lajoie , J. Oyugi , et al., “Differential Elevation of Inflammation and CD4(+) T Cell Activation in Kenyan Female Sex Workers and Non‐Sex Workers Using Depot‐Medroxyprogesterone Acetate,” Frontiers in Immunology 11 (2020): 598307. 33717049 10.3389/fimmu.2020.598307 PMC7949914

[31] K. K. Smith‐McCune , J. F. Hilton , U. Shanmugasundaram , et al., “Effects of Depot‐Medroxyprogesterone Acetate on the Immune Microenvironment of the human Cervix and Endometrium: Implications for HIV Susceptibility,” Mucosal Immunology 10, no. 5 (2017): 1270–1278. 28051087 10.1038/mi.2016.121 PMC5496803

[32] C. Tasker , V. Pizutelli , Y. Lo , B. Ramratnam , N. E. Roche , and T. L. Chang , “Depot Medroxyprogesterone Acetate Administration Increases Cervical CCR5+CD4+ T Cells and Induces Immunosuppressive Milieu at the Cervicovaginal Mucosa,” Aids 34, no. 5 (2020): 729–735. 31972606 10.1097/QAD.0000000000002475 PMC7337252

[33] A. Thurman , N. Chandra , J. L. Schwartz , et al., “The Effect of Hormonal Contraception on Cervicovaginal Mucosal End Points Associated With HIV Acquisition,” Aids Research and Human Retroviruses 35, no. 9 (2019): 853–864. 30997816 10.1089/AID.2018.0298

[34] S. L. Achilles , L. A. Meyn , F. G. Mhlanga , et al., “Zim CHIC: A Cohort Study of Immune Changes in the Female Genital Tract Associated With Initiation and Use of Contraceptives,” American Journal of Reproductive Immunology 84, no. 3 (2020): e13287. 32533883 10.1111/aji.13287 PMC7507197

[35] C. M. Mitchell , L. McLemore , K. Westerberg , et al., “Long‐Term Effect of Depot Medroxyprogesterone Acetate on Vaginal Microbiota, Epithelial Thickness and HIV Target Cells,” Journal of Infectious Diseases 210, no. 4 (2014): 651–655. 24652495 10.1093/infdis/jiu176 PMC4172039

[36] S. Dabee , S. L. Barnabas , K. S. Lennard , et al., “Defining Characteristics of Genital Health in South African Adolescent Girls and Young Women at High Risk for HIV Infection,” PLoS ONE 14, no. 4 (2019): e0213975. 30947260 10.1371/journal.pone.0213975 PMC6448899

[37] A. Weinberg , J. G. Park , R. Bosch , et al., “Effect of Depot Medoxyprogesterone Acetate on Immune Functions and Inflammatory Markers of HIV‐Infected Women,” Journal of Acquired Immune Deficiency Syndromes 71, no. 2 (2016): 137–145. 26413850 10.1097/QAI.0000000000000850 PMC4712075

[38] L. Li , J. Zhou , W. Wang , et al., “Effects of Three Long‐Acting Reversible Contraceptive Methods on HIV Target Cells in the Human Uterine Cervix and Peripheral Blood,” Reproductive Biology and Endocrinology [Electronic Resource]: RB&E 17, no. 1 (2019): 26. 30795774 10.1186/s12958-019-0469-8 PMC6387540

[39] S. L. Achilles , F. G. Mhlanga , P. Musara , S. M. Poloyac , Z. M. Chirenje , and S. L. Hillier , “Misreporting of Contraceptive Hormone Use in Clinical Research Participants,” Contraception 97, no. 4 (2018): 346–353. 28966052 10.1016/j.contraception.2017.09.013 PMC5858917

[40] C. Avenant , A. J. Bick , S. B. Skosana , et al., “Misreporting Contraceptive Use and the Association of Peak Study Progestin Levels With Weight and BMI Among Women Randomized to the Progestin‐Only Injectable Contraceptives DMPA‐IM and NET‐EN,” PLoS ONE 18, no. 12 (2023): e0295959. 38134043 10.1371/journal.pone.0295959 PMC10745193

[41] J. T. Jensen , A. Edelman , C. L. Westhoff , et al., “Use of Serum Evaluation of Contraceptive and Ovarian Hormones to Assess Reduced Risk of Pregnancy Among Women Presenting for Emergency Contraception in a Multicenter Clinical Trial,” Contraception 137 (2024): 110475. 38670302 10.1016/j.contraception.2024.110475 PMC11297684

[42] J. T. Jensen , B. T. Waites , E. R. Boniface , S. McCrimmon , S. Blue , and D. W. Erikson , “Development and Validation of an Expanded Panel of Progestins Using Liquid Chromatography‐Tandem Triple Quadrupole Mass Spectrometry to Monitor Protocol Compliance in Hormonal Contraceptive Pharmacokinetic/Pharmacodynamic Studies,” Contraception 126 (2023): 110130. 37499736 10.1016/j.contraception.2023.110130 PMC10528587

[43] A. N. Nwaohiri , J. H. Tang , F. Stanczyk , et al., “Discordance Between Self‐Reported Contraceptive Use and Detection of Exogenous Hormones Among Malawian Women Enrolling in a Randomized Clinical Trial,” Contraception 97, no. 4 (2018): 354–356. 29246819 10.1016/j.contraception.2017.12.007 PMC5840008

[44] M. Pyra , J. R. Lingappa , R. Heffron , et al., “Concordance of Self‐Reported Hormonal Contraceptive Use and Presence of Exogenous Hormones in Serum Among African Women,” Contraception 97, no. 4 (2018): 357–362. 29408422 10.1016/j.contraception.2018.01.011 PMC5840024

[45] M. Singata‐Madliki , J. Smit , M. Beksinska , et al., “Effects of Injectable Contraception With Depot Medroxyprogesterone Acetate or Norethisterone Enanthate on Estradiol Levels and Menstrual, Psychological and Behavioral Measures Relevant to HIV Risk: The WHICH Randomized Trial,” PLoS ONE 19, no. 3 (2024): e0295764. 38530848 10.1371/journal.pone.0295764 PMC10965066

[46] M. Tomasicchio , C. Avenant , A. Du Toit , R. M. Ray , and J. P. Hapgood , “The Progestin‐Only Contraceptive Medroxyprogesterone Acetate, but Not Norethisterone Acetate, Enhances HIV‐1 Vpr‐Mediated Apoptosis in Human CD4+ T Cells Through the Glucocorticoid Receptor,” PLoS ONE 8, no. 5 (2013): e62895. 23658782 10.1371/journal.pone.0062895 PMC3643923

[47] A. Sivro , A. Schuetz , D. Sheward , et al., “Integrin α(4)β(7) Expression on Peripheral Blood CD4(+) T Cells Predicts HIV Acquisition and Disease Progression Outcomes,” Science Translational Medicine 10, no. 425 (2018): eaam6354. 29367348 10.1126/scitranslmed.aam6354 PMC6820005

[48] J. H. McDonald , Handbook of Biological Statistics, 3rd ed. (Sparky House Publishing, 2014).

[49] K. J. Rothman , “No Adjustments Are Needed for Multiple Comparisons,” Epidemiology (Cambridge, Mass.) 1, no. 1 (1990): 43–46. 2081237

[50] D. J. Saville , “Multiple Comparison Procedures: The Practical Solution,” American Statistician 44 (1990): 174–180.

[51] A. Mohammadi , S. Bagherichimeh , Y. Choi , et al., “Immune Parameters of HIV Susceptibility in the Female Genital Tract Before and After Penile‐Vaginal Sex,” Communications Medicine (London) 2 (2022): 60. 10.1038/s43856-022-00122-7 PMC9142516 35637661

[52] T. Sathaliyawala , M. Kubota , N. Yudanin , et al., “Distribution and Compartmentalization of human Circulating and Tissue‐Resident Memory T Cell Subsets,” Immunity 38, no. 1 (2013): 187–197. 23260195 10.1016/j.immuni.2012.09.020 PMC3557604

[53] O. Ramilo , K. D. Bell , J. W. Uhr , and E. S. Vitetta , “Role of CD25+ and CD25‐T Cells in Acute HIV Infection In Vitro,” Journal of Immunology (Baltimore, Md: 1950) 150, no. 11 (1993): 5202–5208. 8496611

[54] M. Lai‐Goldman , R. K. Nieberg , D. Mulcahy , and E. Wiesmeier , “The Cytobrush for Evaluating Routine Cervicovaginal‐Endocervical Smears,” Journal of Reproductive Medicine 35, no. 10 (1990): 959–963. 2246763

[55] J. M. Gardner , “Analysis of Bleeding Patterns and Resumption of Fertility Following Discontinuation of a Long Acting Injectable Contraceptive,” Fertility and Sterility 21, no. 4 (1970): 286–291. 5508497 10.1016/s0015-0282(16)37443-x

[56] D. R. Mishell Jr. , “Pharmacokinetics of Depot Medroxyprogesterone Acetate Contraception,” Journal of Reproductive Medicine 41, no. 5 Suppl (1996): 381–390. 8725700

[57] D. R. Mishell Jr. , M. A. el‐Habashy , R. G. Good , and D. L. Moyer , “Contraception With an Injectable Progestin. A Study of Its Use in Postpartum Women,” American Journal of Obstetrics and Gynecology 101, no. 8 (1968): 1046–1053. 5663345 10.1016/0002-9378(68)90346-3

[58] S. W. Blue , A. J. Winchell , A. V. Kaucher , et al., “Simultaneous Quantitation of Multiple Contraceptive Hormones in Human Serum by LC‐MS/MS,” Contraception 97, no. 4 (2018): 363–369. 29407362 10.1016/j.contraception.2018.01.015 PMC5840044

[59] R. Heffron , R. Stalter , M. Pyra , et al., “HIV Risk Associated With Serum Medroxyprogesterone Acetate Levels Among Women in East and Southern Africa,” Aids 33, no. 4 (2019): 735–744. 30585845 10.1097/QAD.0000000000002123 PMC6399047

[60] R. P. Molatlhegi , L. J. Liebenberg , A. Leslie , et al., “Plasma Concentration of Injectable Contraceptive Correlates With Reduced Cervicovaginal Growth Factor Expression in South African Women,” Mucosal Immunology 13, no. 3 (2020): 449–459. 31896762 10.1038/s41385-019-0249-y PMC7617771

[61] B. M. Whitney , B. L. Guthrie , S. Srinivasan , et al., “Changes in Key Vaginal Bacteria Among Postpartum African Women Initiating Intramuscular Depot‐Medroxyprogesterone Acetate,” PLoS ONE 15, no. 3 (2020): e0229586. 32134931 10.1371/journal.pone.0229586 PMC7058341

[62] A. K. Ildgruben , I. M. Sjoberg , and M. L. Hammarstrom , “Influence of Hormonal Contraceptives on the Immune Cells and Thickness of Human Vaginal Epithelium,” Obstetrics and Gynecology 102, no. 3 (2003): 571–582. 12962945 10.1016/s0029-7844(03)00618-5

[63] N. Chandra , A. R. Thurman , S. Anderson , et al., “Depot Medroxyprogesterone Acetate Increases Immune Cell Numbers and Activation Markers in Human Vaginal Mucosal Tissues,” Aids Research and Human Retroviruses 29, no. 3 (2013): 592–601. 23189932 10.1089/aid.2012.0271 PMC3581024

[64] Y. Chung and M. J. van der Laan , “Multiple Testing Procedures for High‐Dimensional Data: Review and Comparison,” Statistical Science 26, no. 2 (2011): 180–205.

[65] S. Moran and A. Kor , “False Discovery Rate Control in Small Sample Sizes: A Review of Current Approaches,” Bioinformatics 36, no. 3 (2020): 607–615.

[66] J. P. Shaffer , “Multiple Hypothesis Testing,” Annual Review of Psychology 46 (1995): 561–584.

[67] R. N. Fichorova , P. L. Chen , C. S. Morrison , et al., “The Contribution of Cervicovaginal Infections to the Immunomodulatory Effects of Hormonal Contraception,” MBio 6, no. 5 (2015): e00221–e00315. 26330510 10.1128/mBio.00221-15 PMC4556810

[68] D. Vitali , J. M. Wessels , and C. Kaushic , “Role of Sex Hormones and the Vaginal Microbiome in Susceptibility and Mucosal Immunity to HIV‐1 in the Female Genital Tract,” AIDS Research and Therapy 14, no. 1 (2017): 39. 28893284 10.1186/s12981-017-0169-4 PMC5594427

