# Supplementary material for: Injectable Contraceptives Differentially Affect Select CD4+ HIV‐1 Target Cells in the Genital Tract but Not Systemically: Implications for HIV‐1 Acquisition
Source: Am J Reprod Immunol. 2025 May 11;93(5):e70093. doi: 10.1111/aji.70093 (PMC12066145; doi:10.1111/aji.70093)
Supplement: Supplementary file 2 — Supporting Information [file AJI-93-e70093-s001.docx]

**SUPPLEMENTARY FIGURES**

**
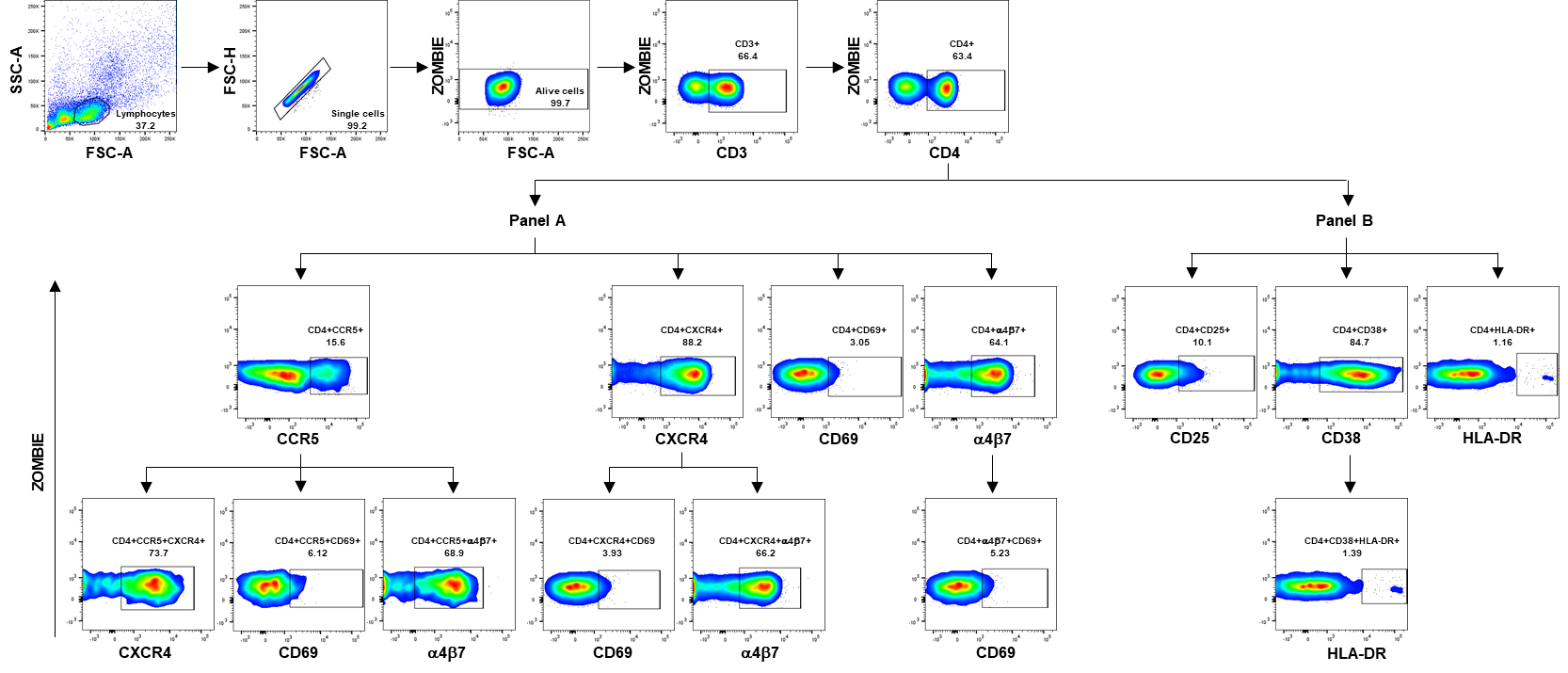
**

**Figure S1. Gating strategy for PBMCs.** PBMCs were stained with two antibody combinations (Panel A and Panel B). For both, lymphocytes were gated from total cells, after which only single cells were analyzed. After gating for live cells (Zombie-), cells were gated for CD3^+^. Thereafter CD3+ populations were gated for CD4^+^ cells. For panel A, CD4^+^ cells were gated for CCR5^+^, CXCR4^+^, CD69^+^ or α4β7^+^ populations, before further gating for double positive CD4^+^ populations (CCR5^+^CXCR4^+^, CCR5^+^CD69^+^, CCR5^+^α4β7^+^, CXCR4^+^CD69^+^, CXCR4^+^α4β7^+^ or α4β7^+^CD69^+^). For panel B, CD4^+^ cells were gated for CD25^+^, CD38^+^ or HLA-DR^+^ populations before further gating for double positive CD4^+^ populations (CD38^+^HLA-DR^+^). Gating was determined using the appropriate fluorescence minus one controls.


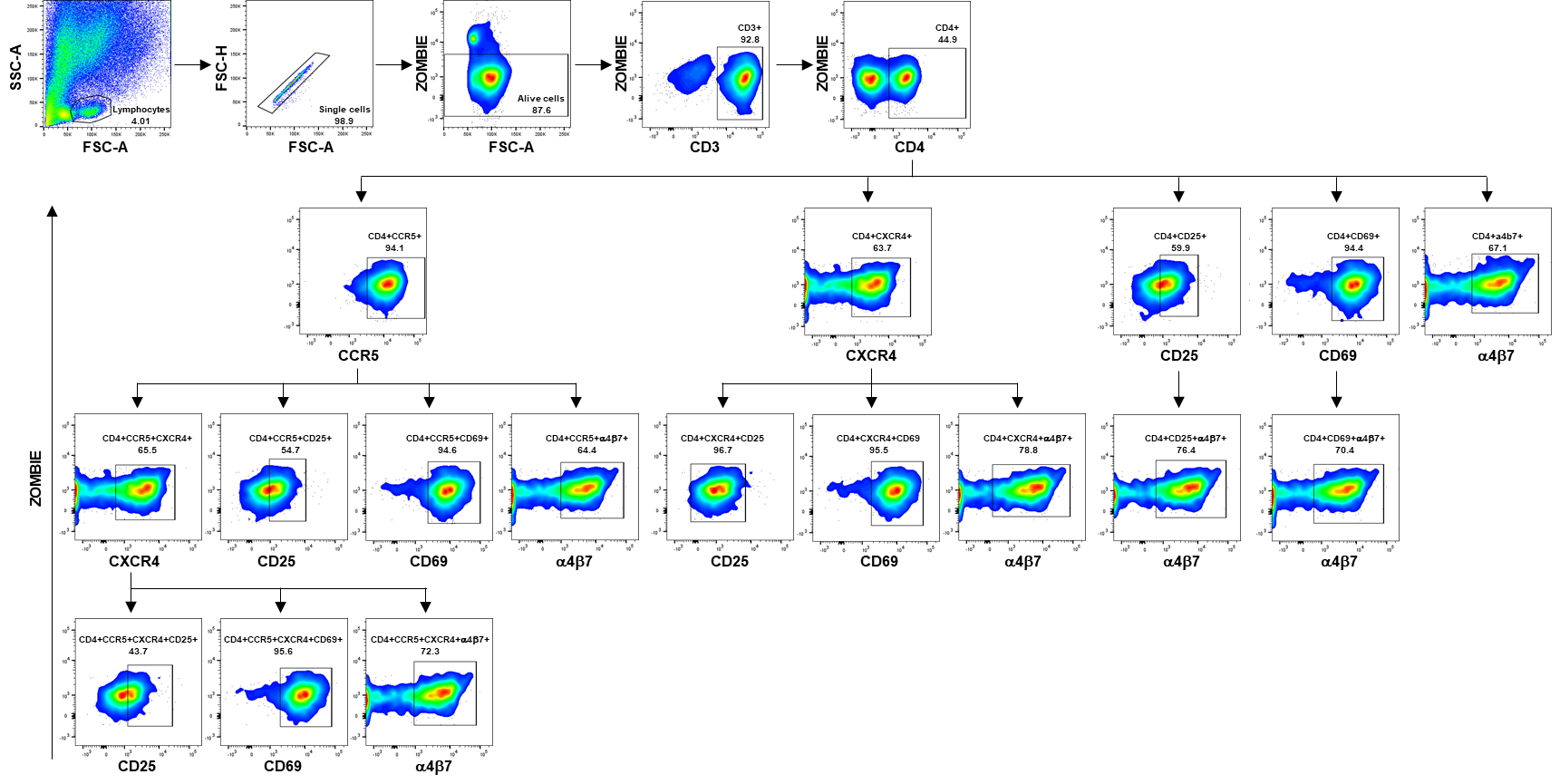


**Figure S2. Gating strategy for cytobrushes.** Lymphocytes were gated from total cells, after which only single cells were analyzed. After gating for live cells (Zombie^-^), cells were gated for CD3^+^. Thereafter CD3^+^ populations were gated for CD4^+^ cells. CD4^+^ cells were then gated for CCR5^+^, CXCR4^+^, CD25^+^, CD69^+^ or α4β7^+^ populations, before further gating for double positive CD4^+^ populations (CCR5^+^CXCR4^+^, CCR5^+^CD25^+^, CCR5^+^CD69^+^, CCR5^+^α4β7^+^, CXCR4^+^CD25^+^, CXCR4^+^CD69^+^, CXCR4^+^α4β7^+^, CD25^+^α4β7^+^ or CD69^+^α4β7^+^. CCR5^+^CXCR4^+^ cells were then further gated for CD25^+^ (CCR5^+^CXCR4^+^CD25^+^), CD69^+^ (CCR5^+^CXCR4^+^CD69^+^) and α4β7^+^ (CCR5^+^CXCR4^+^α4β7^+^) populations. Gating was determined using the appropriate fluorescence minus one controls.
